# Isolates from hospital environments are the most virulent of the *Candida parapsilosis *complex

**DOI:** 10.1186/1471-2180-11-180

**Published:** 2011-08-08

**Authors:** Raquel Sabino, Paula Sampaio, Catarina Carneiro, Laura Rosado, Célia Pais

**Affiliations:** 1 CBMA - Centre of Molecular and Environmental Biology, Department of Biology, University of Minho, Braga, Portugal; 2 Laboratório de Micologia, Departamento de Doenças Infecciosas, Instituto Nacional de Saúde Dr. Ricardo Jorge, Lisboa, Portugal

## Abstract

**Background:**

*Candida parapsilosis *is frequently isolated from hospital environments, like air and surfaces, and causes serious nosocomial infections. Molecular studies provided evidence of great genetic diversity within the *C. parapsilosis *species complex but, despite their growing importance as pathogens, little is known about their potential to cause disease, particularly their interactions with phagocytes. In this study, clinical and environmental *C. parapsilosis *isolates, and strains of the related species *C. orthopsilosis *and *C. metapsilosis *were assayed for their ability to induce macrophage cytotocixity and secretion of the pro-inflammatory cytokine TNF-α, to produce pseudo-hyphae and to secrete hydrolytic enzymes.

**Results:**

Environmental *C. parapsilosis *isolates caused a statistically significant (*p *= 0.0002) higher cell damage compared with the clinical strains, while *C. orthopsilosis *and *C. metapsilosis *were less cytotoxic. On the other hand, clinical isolates induced a higher TNF-α production compared with environmental strains (*p *< 0.0001). Whereas the amount of TNF-α produced in response to *C. orthopsilosis *strains was similar to the obtained with *C. parapsilosis *environmental isolates, it was lower for *C. metapsilosis *strains. No correlation between pseudo-hyphae formation or proteolytic enzymes secretion and macrophage death was detected (*p *> 0.05). However, a positive correlation between pseudo-hyphae formation and TNF-α secretion was observed (*p *= 0.0119).

**Conclusions:**

We show that environmental *C. parapsilosis *strains are more resistant to phagocytic host defences than bloodstream isolates, being potentially more deleterious in the course of infection than strains from a clinical source. Thus, active environmental surveillance and application of strict cleaning procedures should be implemented in order to prevent cross-infection and hospital outbreaks.

## Background

*Candida parapsilosis *is a human commensal of epithelial and mucosal tissues, also frequently isolated from hospital environments, like air and surfaces. It is the cause of serious nosocomial infections, being the second most common fungal species isolated from blood in many regions of the world [1-3]. Due to its association with parenteral nutrition and intravascular catheters, *C. parapsilosis *affects mainly critically ill patients from surgical intensive care units, neonates, and cancer patients [4-6]. Neonates are especially prone to candidemia, and in low weight infants the estimated incidence of invasive infections due to *C. parapsilosis *is 2%, reaching as much as 10% in extreme cases [7-9].

The modes of transmission and portals of entry of fungal nosocomial infections vary according to the pathogen involved. *Candida *infections are predominantly of endogenous origin but cross-infection via hands of health care workers or relatives, or through devices has been shown to occur [10]. Invasive fungal infections may be acquired in the hospital from different sources, and numerous fungal reservoirs have been identified in hospital environment, including unfiltered air, ventilation systems, contaminated dust during hospital construction, carpeting, water, food, and ornamental plants [11]. In fact, environmental exposure to *C. parapsilosis *from hospital healthcare workers has been associated with both sporadic cases and outbreaks of invasive fungal infections in immunocompromised patients [12,13].

Most pathogenic *Candida *species have developed a wide range of putative virulence factors to assist in their ability to colonize host tissues, cause disease, and overcome host defenses. Among them, secretion of hydrolytic enzymes such as aspartic proteinases and lipases, as well as morphogenesis have been well studied in *C. albicans *[14-16]. However, despite the growing importance of the *C. parapsilosis *species complex, few works evaluating the *in vitro *virulence of these species have been performed [17-19] and little is known about the virulence traits that enable them to cause disease.

Mononuclear phagocytes play an important role in innate immunity, in the polarization of the immune adaptive response and also in the eradication of *Candida *sp. [20,21]. Given the critical role played by macrophages in balancing colonization/infection, the analysis of their interaction with isolates belonging to the *C. parapsilosis *complex is important to understand the virulence potential of these species.

In the present work, we compared *C. parapsilosis *bloodstream isolates and strains recovered from the hospital setting regarding their virulence in vitro. Mononuclear phagocytes were used to test the strain ability to: (i) induce cytotocixity; (ii) activate TNF-α release; (iii) filament *in vitro*, both during macrophage infection and in the presence of serum, and (iv) secrete hydrolytic enzymes. *Candida parapsilosis *environmental isolates revealed to be the most virulent to macrophage cells, being potentially more deleterious, particularly in the initial phases of the infection, than strains from a clinical source.

## Results

### *Candida parapsilosis *interaction with macrophages

**Figure 1:**
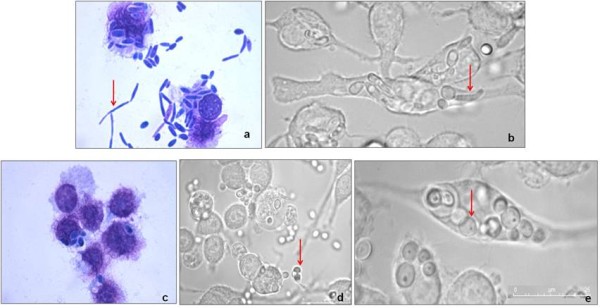
**Microscopic observations of *C. parapsilosis *incubated with J774 macrophages**. Hemacolor staining and bright field images of the co-incubation of macrophages with the clinical isolate 972697 (a and b) and the environmental isolate CarcC (c to e), after 12 hours. Arrows point to the different yeast morphologies in contact with macrophages.

The ability of macrophages to kill *C. parapsilosis *bloodstream isolates and environmental strains was determined by CFU counting after one hour co-incubation, using six isolates of each. The average percentage of yeast killing for the environmental isolates was 10.97 ± 2.67 while for clinical isolates it was 33.22 ± 5.25, the difference being statistically significant (*p *= 0.0409). The interaction of one clinical and one environmental isolate with macrophages was followed for 12 hours of incubation. Microscopic examination showed that the clinical isolate was able to produce pseudo-hyphae and maintained that ability in contact with macrophages (Figure 1a and 1b), while the environmental isolate kept the yeast unicellular morphology (Figure 1c to 1e).

**Figure 2:**
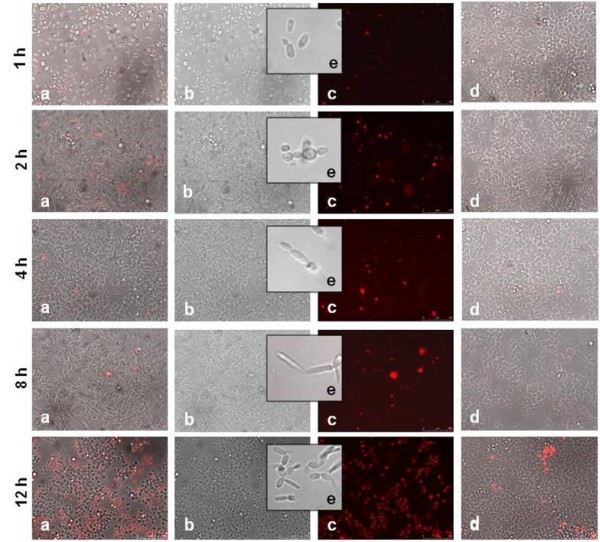
**Macrophage death upon contact with the *C. parapsilosis *blood isolate 972697**. To detect dead phagocytes, cells were incubated with 1 μg/ml of PI and visualized at 1, 2, 4, 8, and 12 hours of infection with a magnification of 200 ×. Necrotic nuclei are presented in red. Images were obtained using fluorescent microscopy (c), bright-field microscopy (b) and the superposition of both (a). At each time point, dead macrophages without *C. parapsilosis *served as negative control (d). *C. parapsilosis *cell morphology in the absence of macrophages (e).

**Figure 3:**
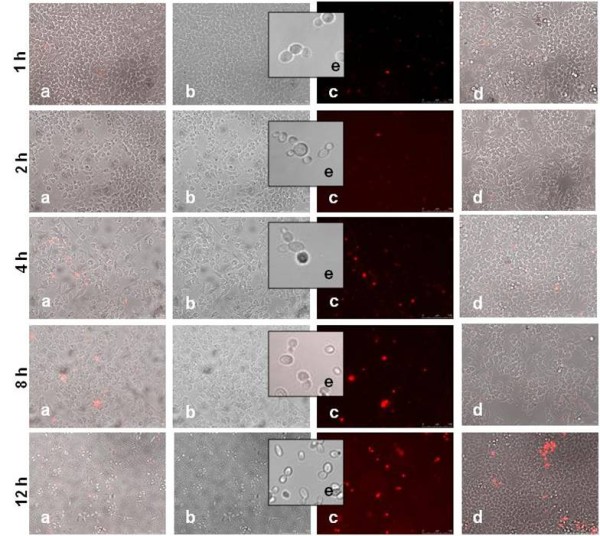
**Macrophage death upon contact with the *C. parapsilosis *environmental strain CarcC**. To detect dead phagocytes, cells were incubated with 1 μg/ml of PI and visualized at 1, 2, 4, 8, and 12 hours of infection with a magnification of 200 ×. Images were obtained using fluorescent microscopy (c), bright-field microscopy (b) and the superposition of both (a). At each time point, dead macrophages without *C. parapsilosis *served as negative control (d). *C. parapsilosis *cell morphology in the absence of macrophages (e).

The percentage of dead macrophages after co-incubation with the same two isolates, assessed by propidium iodide (PI) staining, showed that macrophage killing did not vary significantly in the first 8 hours of incubation, with percentages of macrophage death similar to the negative control (Figure 2 and 3). However, after 12 hours of infection with the clinical isolate the percentage of macrophage killing increased to 41% (Figure 2c, 12 h). On the contrary, after 12 hours co-incubation with the environmental strain, the number of macrophages in the slide was significantly reduced (Figure 3a, b, 12 h) when compared with the first hours of infection, and with the negative control (Figure 3d, 12 h) and many yeast cells could be observed. Therefore, in this case, the proportion of PI positive cells could not be quantified due to the reduction of macrophage cell numbers, probably by cell lysis. Together, these observations suggested that clinical and environmental isolates behave differently in contact with macrophages. Although unable to filament, the environmental isolate induced macrophage cytotoxicity and cell lysis, which is consistent with the lower yeast killing observed previously. Following these results, twenty-five blood isolates and twenty environmental isolates were selected to test these findings, and the studies were extended to eight *C. orthopsilosis *and four *C. metapsilosis *strains, for comparison.

### *Candida parapsilosis *environmental isolates are more cytotoxic to macrophages

**Figure 4:**
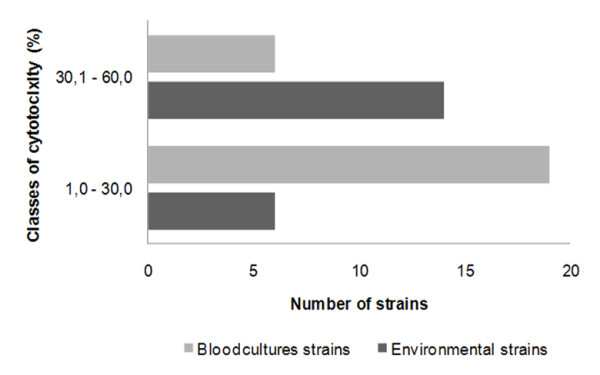
**Distribution of *C. parapsilosis *environmental and blood isolates, according to the defined classes of cytotoxicity**. The majority of environmental isolates are included in the group causing between 30 and 60% cytotoxicity. Cellular damage induced by the yeast was quantified as the amount of LDH release by macrophages after 12 hours of infection.

The release of LDH by macrophages was monitored after 12 hours of co-incubation using all the different strains analysed in this study (Table 1). Results showed that the percentage of cytotoxicity varied from 6.4% to 59.2%, revealing a great variability in strain ability to induce damage. Due to this variability the isolates were grouped into two classes of cytotoxicity and it was observed that the great majority of environmental strains exhibited cytotoxicity levels between 30.1 and 60.0%, while clinical isolates were mainly in the group presenting 1 to 30% cytotoxicity (Figure 4). Overall, the environmental isolates induced statistically significant (*p *< 0.0001) higher cell damage (average 37.6% ± 13.78) when compared with the clinical strains (22.9 ± 10.36). Regarding *C. orthopsilosis *and *C. metapsilosis *the average percentage of induced cytotoxicity was 19.3% (± 6.17) and 8.8% (± 1.05), respectively.

**Table 1:** Species used in this study, their collection date, and origin

	Species	Isolate identification	Geographical origin	Collection date	Product
**Environmental**	*C. parapsilosis*	IPOA1	Portugal - Hospital 1	2007	Water tap nursery 23
	*C. parapsilosis*	IPOA2	Portugal - Hospital 1	2007	Bedside table no. 4 nursery 30
	*C. parapsilosis*	IPOA3	Portugal - Hospital 1	2007	Water tap nursery 24
	*C. parapsilosis*	IPOA14	Portugal - Hospital 1	2007	Treatment room
	*C. parapsilosis*	IPOA15	Portugal - Hospital 1	2007	Door knob Patients' WC
	*C. parapsilosis*	IPOA20	Portugal - Hospital 1	2007	Air from individual room no.5
	*C. parapsilosis*	IPOA21	Portugal - Hospital 1	2007	Water tap treatment room
	*C. parapsilosis*	IPOA22	Portugal - Hospital 1	2007	Shower Patients' WC
	*C. parapsilosis*	IPOA23	Portugal - Hospital 1	2007	Air from nursery 24
	*C. parapsilosis*	CNR40	France	2007	Hospital environment
	*C. parapsilosis*	494F	France	2007	Hospital environment
	*C. parapsilosis*	Carc	Portugal	2006	Beach sand
	*C. parapsilosis*	Avc	Portugal	2006	Beach sand
	*C. parapsilosis*	Pr b	Portugal	2006	Beach sand
	*C. parapsilosis*	1144	Portugal - Hospital 2	2006	Hospital air
	*C. parapsilosis*	1156	Portugal - Hospital 2	2006	Hospital air
	*C. parapsilosis*	1159	Portugal - Hospital 2	2006	Hospital air
	*C. parapsilosis*	1160	Portugal - Hospital 2	2006	Hospital air
	*C. parapsilosis*	1182	Portugal - Hospital 2	2006	Hospital air
	*C. parapsilosis*	1194	Portugal - Hospital 2	2006	Hospital air

**Clinical**	*C. parapsilosis*	376604	Portugal - Hospital 1	2002	Blood culture
	*C. parapsilosis*	378058	Portugal - Hospital 1	2002	Blood culture
	*C. parapsilosis*	378690	Portugal - Hospital 1	2002	Blood culture
	*C. parapsilosis*	433573	Portugal - Hospital 1	2003	Blood culture
	*C. parapsilosis*	431472	Portugal - Hospital 1	2003	Blood culture
	*C. parapsilosis*	476446	Portugal - Hospital 1	2003	Blood culture
	*C. parapsilosis*	506858	Portugal - Hospital 1	2003	Blood culture
	*C. parapsilosis*	522760	Portugal - Hospital 1	2004	Blood culture
	*C. parapsilosis*	864647	Portugal - Hospital 1	2006	Blood culture
	*C. parapsilosis*	814455	Portugal - Hospital 1	2006	Blood culture
	*C. parapsilosis*	972697	Portugal - Hospital 1	2007	Blood culture
	*C. parapsilosis*	20L	France	2004	Blood culture
	*C. parapsilosis*	155	France	2004	Blood culture
	*C. parapsilosis*	202	France	2004	Blood culture
	*C. parapsilosis*	272	France	2004	Blood culture
	*C. parapsilosis*	465	France	2005	Blood culture
	*C. parapsilosis*	573	France	2005	Blood culture
	*C. parapsilosis*	648	France	2006	Blood culture
	*C. parapsilosis*	899	France	2006	Blood culture
	*C. parapsilosis*	CAN16	Portugal - Hospital 3	2002	Blood culture
	*C. parapsilosis*	CAN159	Portugal - Hospital 3	2004	Blood culture
	*C. parapsilosis*	CAN201	Portugal - Hospital 3	2005	Blood culture
	*C. parapsilosis*	CAN270	Portugal - Hospital 3	2006	Blood culture
	*C. parapsilosis*	CAN279	Portugal - Hospital 3	2007	Blood culture
	*C. parapsilosis*	H1	USA	-	Blood culture
	*C. ortopsilosis*	754	Portugal - Hospital 2	2004	Bronchial secretions
	*C. ortopsilosis*	755	Portugal - Hospital 2	2004	Bronchial secretions
	*C. ortopsilosis*	892	Portugal - Hospital 2	2004	Blood culture
	*C. ortopsilosis*	894	Portugal - Hospital 2	2004	Blood culture
	*C. ortopsilosis*	895	Portugal - Hospital 2	2004	Blood culture
	*C. ortopsilosis*	981224	USA	-	Unknown
	*C. ortopsilosis*	H10	USA	-	Unknown
	*C. ortopsilosis*	CAN 138	Portugal - Hospital 3	2004	Blood culture
	*C. metapsilosis*	911012	Portugal - Hospital 1	2006	Blood culture
	*C. metapsilosis*	CAN 155	Portugal - Hospital 3	2004	Blood culture
	*C. metapsilosis*	960161	USA	-	Unknown
	*C. metapsilosis*	am 2006	USA	-	Unknown

### Clinical isolates of *C. parapsilosis *are able to induce a higher inflammatory response in infected macrophages

**Figure 5:**
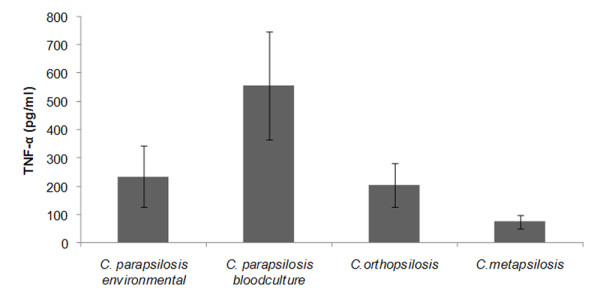
**Determination of TNF-α release**. Level of TNF-α release by macrophages infected with environmental and bloodculture *C. parapsilosis *isolates, and with *C. orthopsilosis*, and *C. metapsilosis *isolates after 12 hours of infection.

The amount of TNF-α released by infected macrophages was quantified as an indication of the yeast potential to induce an inflammatory response. TNF-α released varied from 50.51 to 809.4 pg/ml (Figure 5). The blood isolates induced a higher TNF-α secretion (average 557.7 ± 190.95 pg/ml) compared with the environmental strains (average 234.6 ± 108.7 pg/ml) and this difference was statistically significant (*p *< 0.0001). The average amount of TNF-α production by *C. orthopsilosis *strains was 204.6 ± 77.40 pg/ml, similar to *C. parapsilosis *environmental isolates, whereas for *C. metapsilosis *only 75.4 ± 23.84 pg/ml was detected. All comparisons were statistically significant (*p *< 0.05) except for *C. orthopsilosis *vs environmental *C. parapsilosis *strains.

### Pseudo-hyphae formation and secretion of aspartic proteinase and phospholipase

Virulence factors such as secretion of hydrolytic enzymes, aspartic proteinases and/or phospholipases, and pseudo-hyphae formation are likely to contribute to *Candida *cytotoxicity. These characteristics were measured in all isolates used in this study and results are shown in Table 2. About 60% of *C. parapsilosis *isolates were able to produce pseudo-hyphae after 12 hours of incubation. Interestingly, comparing environmental with clinical isolates, the majority of the pseudo-hyphae producers were the clinical ones, and this difference was statistically significant (χ^2 ^= 4.664, *p *= 0.0154). Around half of the *C. orthopsilosis *strains produced pseudo-hyphae, while none of the *C. metapsilosis *isolates was able to filament.

High proteinase activity was found in 36 (80.0%) *C. parapsilosis *strains, being 38.8% environmental and 61.2% clinical isolates (Table 2). However, no significant difference (χ^2 ^= 2.250, *p *= 0.0688) was observed when comparing environmental and clinical isolates. No Sap production was observed in most of the *C. orthopsilosis *and *C. metapsilosis *isolates (Table 2). No significant phospholipase production was detected in the tested isolates.

**Table 2:** Pseudo-hyphae and secreted aspartyl proteinase (sap) production

Isolates	Pseudo-hyphae production		Sap production	
	
	Yes	No	High	Low
*C. parapsilosis *				
Environment	8	12	14	6
Bloodcultures	18	7	22	3
*C. orthopsilosis*	3	5	2	6
*C. metapsilosis*	0	4	0	4
Total no. of strains	29	28	38	19

Study of 45 environmental and clinical *Candida parapsilosis *isolates, 8 *C. orthopsilosis *and 4 *C. metapsilosis *strains.

## Discussion

*Candida parapsilosis *accounts for a significant proportion of nosocomial infections, with an increasing prevalence in hospital settings. As with other *Candida *species, invasion of *C. parapsilosis *can result in severe disease, particularly in hosts with a compromised immune system. Unlike *C. albicans*, the transmission and acquisition of infection due to *C. parapsilosis *is mainly exogenous and environmental strains are often the source of infection. The main issue of this study was, therefore, the comparison of the virulence potential of environmental and clinical *C. parapsilosis *isolates.

Macrophages play an important role in the immune response, directly by phagocytosing and killing microbial pathogens, and indirectly by processing and presenting antigens and secreting cytokines [22]. Although there were variations in the intracellular killing of the different strains, the average percentage was of about 35% for the clinical isolates, in agreement with the results obtained by Gácser *et al*. [18] for *C. parapsilosis*. Curiously, these values were much lower for the environmental strains, pointing to a clear difference between environmental and clinical isolates, regarding interaction with macrophages. A great variability in the capacity of the strains to cause cell damage was also found, and again environmental isolates induced significantly higher macrophage damage than blood isolates, confirming a strong relationship between the source of the isolates and their ability to cause damage. It was also observed that *C. orthopsilosis *induced a high level of macrophage damage, similar to *C. parapsilosis *bloodstream isolates, while *C. metapsilosis *induced the lowest cytotoxicity level. These facts agree with previous works on reconstituted human oral epithelial and epidermal tissues [19] and microglial cells [23], showing that *C. metapsilosis *was less virulent compared to *C. orthopsilosis *and *C. parapsilosis*. To correlate these findings with the morphology, yeast strains were induced to filament in the presence of serum and results showed that 57.7% of the tested *C. parapsilosis *isolates were able to produce pseudo-hyphae after 12 hours of incubation, with the clinical isolates filamenting in a higher percentage than the environmental strains. Curiously, this high filamentation ability was not correlated with higher macrophage cytotoxicity as it has been described for *C. albicans *[24,25]. In our study, although *C. parapsilosis *filamentation occurred right after 4 hours, differences in macrophage death were observed only after 12 hours of co-incubation. Incubation with the strains that did not develop pseudo-hyphae revealed that, after 12 hours of infection, a huge number of macrophages had disappeared and the yeast number was high. This observation could be explained by the hypothesis that yeast replicate inside macrophages and lyse them, enabling the release of yeast into the medium, as well as LDH. Since filamentation was not responsible for the death of the macrophages incubated with the environmental strains, maybe other virulence factors could account for these observations.

Secretion of hydrolytic enzymes such as aspartic proteinases and phospholipases have been associated with *C.albicans *virulence [14,16,26,27] and also with *C. parapsilosis *virulence [15,18,28-31]. Eighty percent of the tested *C. parapsilosis *strains were found to have high proteinase activity, being the majority blood isolates. To our knowledge, no other study compared Sap production in clinical and environmental *C. parapsilosis *isolates, but Dagdeviren et al. [32] observed a higher production of acid proteinase among *C. parapsilosis *blood isolates compared to non-blood isolates. From the eight *C. orthopsilosis *tested only 25% were Sap producers, whereas none of the *C. metapsilosis *was. This is in accordance with Lin et al. [33], who also reported differences in proteinase activity within the three major groups of *C. parapsilosis*.

No correlation was observed between hydrolytic enzymes secretion and environmental or clinical isolates, or with cell damage (p > 0.05).

Macrophage activation induces releasing of several key mediators, including proinflammatory cytokines such as TNF-α, which are important for protecting the host against disseminated candidiasis [34-36]. The amount of TNF-α produced by macrophages infected with *C. parapsilosis *isolates from bloodcultures was significantly higher than the amount produced by macrophages infected with environmental isolates, indicating that clinical isolates induce a higher pro-inflammatory response than environmental strains. The fact that a high macrophage cell lysis occurred in the co-incubations with the environmental strains could also account for these results. In contrast, Orsi et al. [23] reported little or no TNF-α production in the co-incubations of strains of the *C. parapsilosis *complex with microglial cells. This discrepancy may result from the fact that the 6-hour incubation time used in their study was insufficient to trigger cell response. Our results showed a positive correlation between filamentation and TNF-α release (p = 0.0119) for *C. parapsilosis. Candida orthopsilosis *strains induced TNF-α levels similar to the clinical isolates, whereas *C. metapsilosis *isolates induced the production of lower amounts, which is in agreement with Gácser et al. [19] who showed that *C. metapsilosis *appears as the less virulent of the three species of the *C. parapsilosis *complex. Nevertheless, recent literature indicates that *C. metapsilosis *can be retrospectively identified at a frequency similar to *C. orthopsilosis *and from virtually all body sites [37,38]. In addition, a meta-genomic study has found *C. metapsilosis *sequences in the oral cavity of healthy carriers, suggesting the possibility of oral commensalism for this species [39].

## Conclusions

Overall, this report evidences for the first time that environmental and clinical *C. parapsilosis *isolates behave differently in contact with macrophages, indicating that environmental strains cause a higher cellular damage and seem to be more prone to resist to macrophage killing. Since nosocomial fungal infections progress rapidly, and *C. parapsilosis *is frequently isolated from the hospital settings, there is a critical need for more efforts toward prevention, early diagnosis, and effective treatment of these infections. Among the preventive measures the environmental surveillance and strict application of cleaning procedures are of major importance to prevent the onset of hospital outbreaks.

## Methods

### *Candida *isolates and preparation of cell suspensions

Forty-five *C. parapsilosis *isolates, eight *C. orthopsilosis *isolates, and four *C. metapsilosis *isolates were used in this study (Table 1). Twenty-five of the *C. parapsilosis *isolates were from bloodstream infections, and 20 were obtained from the hospital environment, including bedside tables, doors knobs, surfaces, and air. The identity of the isolates was confirmed at the species level by locus specific amplification [40] or by sequencing the ribosomal ITS region [41]. Yeast cells were grown overnight at 37°C in YEPD medium (2% glucose, 1% bacto peptone, and 2% yeast extract), recovered by centrifugation, washed in sterile PBS buffer, and a suspension of 2 × 10^7^cells/ml was prepared in Dulbecco's Modified Eagle's Medium (DMEM).

### Macrophage culture and determination of candidacidal activity

The murine macrophage-like cell line J774A.1 (American Type Culture Center number TIB 67Ralph and Nakoinz, 1975) was cultured in complete DMEM supplemented with 10% heat-inactivated fetal calf serum (FBS), at 37°C in a 5% CO_2 _atmosphere. After confluent growth, macrophage cells were recovered, washed, and re-suspended in DMEM to a final concentration of 4 × 10^5^cells/ml. Yeast killing was assessed by using a multiplicity of infection (MOI) of 1:10 in 24 well tissue-culture plates (Orange) for 60 minutes, at 37°C in a 5% CO_2 _atmosphere. After incubation macrophage cells were lysed with 800 μl of cold water and wells scrapped to ensure removal of all the yeast cells. Lysates were serially diluted and plated on YEPD agar to determine the percentage of viable yeast cells. Controls consisted of yeast cells grown in the same conditions but without macrophages. Candidacidal activity (%) was calculated using the following formula: [(CFU of control well - CFU of test well)/CFU of control well] × 100. Each strain was tested in triplicate.

### Analysis of *C. parapsilosis *morphology during macrophage infection

Yeast cell morphology in contact with macrophages was evaluated by co-incubating the macrophage cell line with *Candida *cells, as described above. Macrophage cells were seeded into 24 well tissue-culture plates containing a plastic coverslip in each well (Nunc, Rochester, USA) to allow macrophage adherence. After 1, 4, 8, 10 and 12 hours of co-incubation coverslips were removed, fixed with 10% formol on ethanol, and Hemacolor (Merck, New Jersey, EUA) stained. Different fields were analyzed under a Leica DM5000B light microscope and images captured with a Leica DFC350FX camera.

### Macrophage death assessment

Kinetic of macrophage death was assessed by incubating macrophages with *C. parapsilosis *at a MOI of 1:10 as previously described. Macrophage death was assayed by determining the percentage of cells with plasma membranes permeable to propidium iodide (PI) after 1, 2, 3, 4, 6, 8, 10 and 12 hours of co-incubation. Cells on the coverslips were stained with 1 μg/ml PI at room temperature for 10 min in the dark, and observed using a Leica DM5000B fluorescence microscope. At each time point, images were taken and approximately 1000 cells were counted in independent fields. The percentage of macrophage cells permeable to PI was calculated as described by Shin *et al*. [24].

### Lactate dehydrogenase (LDH) measurement

The release of LDH from cells into the medium was monitored as a measure of cell damage. LDH released in the medium from macrophage cultures (negative control) and from macrophages co-incubated with *C. parapsilosis, C. orthopsilosis and C. metapsilosis *was measured after 12 h incubation by using the Cytotoxicity Detection Kit PLUS (LDH) (Roche Diagnostics Corporation, Indianapolis, USA), according to the manufacturer's instructions.

### Cytokine measurement

TNF-α production by macrophages infected with the strains in study was measured using the Mouse TNFα ELISA ReadySETGoKit (eBioscience, San Diego, CA, USA), according to the manufacturer's instructions.

### Secreted aspartic proteinase and phospholipase production

The production of secreted aspartic proteinases (Sap) and phospholipases by isolates of *C. parapsilosis, C. orthopsilosis *and *C. metapsilosis *was determined as previously described [42]. One *C. albicans *producer strain (SC5314) was added as a positive control.

### Filamentation assay

Filamentation was assessed by seeding 200 μl of the prepared cell suspensions into 24 well tissue-culture plates (Orange), and incubating at 37°C in a 5% CO2 atmosphere for 12 hours. An aliquot of each suspension was then smeared onto a glass slide and images were taken with a Leica DM5000B light microscope.

### Statistical analysis

Unless otherwise stated, results shown are the mean of three independent experiments ± SD. Statistical significance of results was determined by the T student test or the χ^2^-test. Results were considered statistically significant when two-tailed p values were less than 0.05. All calculations were performed with GraphPad Prism 5 software.

## Authors' contributions

PS and CP conceived and designed the study. RS, PS, and CC performed the experiments; RS, PS, LR, and CP analyzed the data; RS, PS and CP wrote the manuscript. All authors have read and approved the final version of the manuscript
